# Proteomic Investigation of Cape Cobra (*Naja nivea*) Venom Reveals First Evidence of Quaternary Protein Structures

**DOI:** 10.3390/toxins16020063

**Published:** 2024-01-23

**Authors:** Lewis O. McFarlane, Tara L. Pukala

**Affiliations:** Department of Chemistry, The University of Adelaide, Adelaide 5005, Australia; lewis.mcfarlane@adelaide.edu.au

**Keywords:** venom, snake, *N. nivea*, Cape Cobra, proteomics, protein complex, 3FTx, PLA_2_

## Abstract

*Naja nivea* (*N. nivea*) is classed as a category one snake by the World Health Organization since its envenomation causes high levels of mortality and disability annually. Despite this, there has been little research into the venom composition of *N. nivea*, with only one full venom proteome published to date. Our current study separated *N. nivea* venom using size exclusion chromatography before utilizing a traditional bottom-up proteomics approach to unravel the composition of the venom proteome. As expected by its clinical presentation, *N. nivea* venom was found to consist mainly of neurotoxins, with three-finger toxins (3FTx), making up 76.01% of the total venom proteome. Additionally, cysteine-rich secretory proteins (CRISPs), vespryns (VESPs), cobra venom factors (CVFs), 5′-nucleotidases (5′NUCs), nerve growth factors (NGFs), phospholipase A2s (PLA_2_), acetylcholinesterases (AChEs), Kunitz-type serine protease inhibitor (KUN), phosphodiesterases (PDEs), L-amino acid oxidases (LAAOs), hydrolases (HYDs), snake venom metalloproteinases (SVMPs), and snake venom serine protease (SVSP) toxins were also identified in decreasing order of abundance. Interestingly, contrary to previous reports, we find PLA_2_ toxins in *N. nivea* venom. This highlights the importance of repeatedly profiling the venom of the same species to account for intra-species variation. Additionally, we report the first evidence of covalent protein complexes in *N. nivea* venom, which likely contribute to the potency of this venom.

## 1. Introduction

In 2017, the World Health Organisation (WHO) made the controversial decision to add snake bite envenomation (SBE) to their list of neglected tropical diseases, making SBE the only non-infectious disease on the list [1,2]. SBE has more estimated deaths per year (8000–120,000) than any other neglected tropical disease on the WHO list [2]. Of those deaths per annum, at least 30,000 of them occur in Africa, with most occurring in rural areas where access to basic health care is extremely limited [3,4]. Of the 400 snake species in Africa, 135 are venomous and are considered to be medically relevant by WHO [5]. These 135 snakes are designated into significance categories of 1–5 depending on the frequency at which their bite is associated with life-threatening envenomation, with category 1 snakes being the most likely to cause a fatal envenomation [6]. Cobras, both spitting and non-spitting, make up the majority of category 1 snakes due to their potent venom, aggression and, in some cases, their tendency to enter human settlements [6]. One of these cobras is the Cape Cobra, *Naja nivea* (*N. nivea*), a non-spitting cobra that is found largely in the Cape of South Africa [6,7,8,9,10,11,12,13,14]. *N. nivea* envenomation is often fatal due to the rapid onset of respiratory paralysis, a symptom that can be attributed to the large number of small neurotoxins found in this venom [14,15]. Despite its high level of medical importance, there has been a lack of research into the venom of *N. nivea*, with the majority of studies being single toxin studies from the 1970s–1980s [15,16,17,18,19,20]. In 2022, Tan et al. [14]. published the first full venom proteome of *N. nivea*; however, due to well-reported differences in venom proteomes within snakes of the same species, it is important to continue efforts to characterise *N. nivea* venom to gain a more thorough understanding of its proteome [8,9,10,11,12,13,14]. Contrary to the approach by Tan et al. [14], we utilized an in-gel digestion approach in preference to in solution in order to increase the coverage of our proteomic analysis.

It is widely understood that in order to decrease the global burden of SBE, improvements need to be made to our current treatment methods [21]. The only effective treatment of SBE is the use of antivenom, which consists of immunoglobins raised to target specific snake venoms [22]. This method of SBE treatment comes with a myriad of issues that contribute to the global burden of SBE. Antivenom has very poor stability and frequent adverse reactions, and it is often too expensive for the people who need it most [21,23]. Additionally, the immunoglobins in antivenom are often raised to target a mixture of several different venoms, for example, SAIMR Polyvalent Antivenom, which was raised against 10 species of venomous snakes, including *N. nivea* [24]. This presents issues as the venom proteome of snakes varies drastically between species, resulting in varied treatment efficacies depending on which toxins the immunoglobins are raised to neutralise. In order to design alternative antivenom treatments there first needs to be a greater understanding of the venom proteome of all medically relevant snakes. This will allow researchers to find conserved venom toxins to use as targets for the design of alternative treatment methods.

Snake venom proteomes are made up of a complex mixture of enzymes, non-enzymatic proteins and peptides that have evolved to aid in the immobilisation and digestion of prey [25]. The enzymatic constituents consist of a range of toxin families, such as phospholipase A_2_s (PLA_2_s), snake venom serine proteases (SVSPs), snake venom metalloproteinases (SVMPs), L-amino acid oxidases (LAAOs), acetylcholinesterases (AChEs), 5′-nucleotidases (5′-NUCs), phosphodiesterases (PDEs), hydrolases (HYDs), and L-amino acid oxidases (LAAOs) [25]. These toxins are known to disrupt cellular processes such as haemostasis [25,26]. Non-enzymatic toxins are equally potent and largely associated with neurological and cardiovascular effects [27]. There are a large number of non-enzymatic toxin families including 3-finger toxins (3FTx), C-type lectins (CTLs), nerve growth factors (NGFs), cysteine-rich secretory proteins (CRISPs), cobra venom factors (CVFs), vespryns (VESPs), and Kunitz-type serine protease inhibitor (KUN) [28]. In addition to their individual toxic properties, both enzymatic and non-enzymatic venom toxins have been known to form higher-order structures that enhance the potency of the venom [29,30,31,32,33]. For example, a paper by Wang et al. investigated the activity of monomeric and dimeric PLA_2_ enzymes and found a notable increase in activity for the dimeric form [33]. This suggests that higher-order structures increase the potency of venom and hence, it is vital that quaternary structures are considered when initially profiling a snake’s venom proteome. Here, we present an in-depth characterisation of *N. nivea* venom proteome and the first evidence of quaternary structures in this species’ venom.

## 2. Results and Discussion

### 2.1. Fractionation with Size Exclusion Chromatography

**Figure 1:**
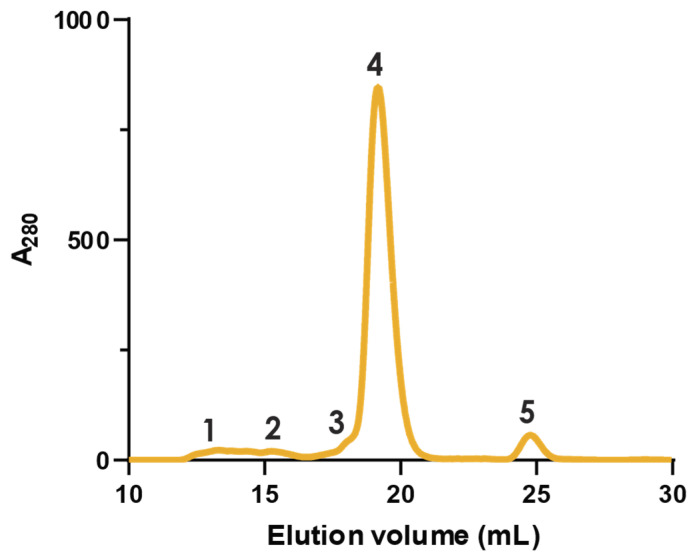
Size exclusion chromatography elution profile of pooled whole venom from *N. nivea* eluted in 200 mM ammonium acetate. Peaks are labelled in order of elution.

**Figure 2:**
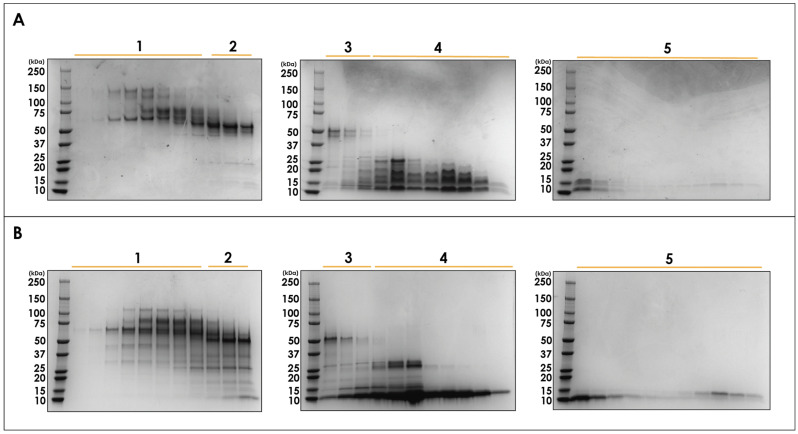
Non-reduced (**A**) and reduced (**B**) SDS-PAGE analysis of *N. nivea* venom. Fractions corresponding to pooled size exclusion peaks are indicated for reference.

Crude pooled *N. nivea* venom was fractionated using size exclusion chromatography (SEC) to obtain a general view of venom complexity (Figure 1). Proteins were eluted in ammonium acetate buffer, which maintains their native conformation, ensuring any non-covalent protein complexes are kept intact [34], and is compatible with downstream mass spectrometry where relevant. SEC separation yielded five distinct peaks, labelled in order of elution, over a 30 mL elution volume with the largest protein species eluting first. It can be seen that this venom contains principally small-molecular-weight proteins or protein complexes with a handful of larger species (Figure 2). This is consistent with expectations due to the neurotoxic properties of *N. nivea* venom and the size of known neurotoxic venom proteins, which tend to be in the 8–5 kDa range [15,25,27,35].

To gain further insights into the components of *N. nivea* venom and investigate the presence of covalent protein complexes, non-reducing and reducing SDS-polyacrylamide gel electrophoresis (SDS-PAGE) experiments were performed on the collected SEC fractions (Figure 2). Comparing the bands from the reducing and non-reducing conditions allows us to gain insights into any protein complexes linked covalently by disulphide bonds within this venom. When comparing the gels of all five SEC peaks, it can be seen that the electrophoretic profiles of each peak differ drastically between conditions. Interestingly, every collected SEC fraction contains at least one covalent protein complex, as evidenced by the significant reduction in molecular weights observed for the components upon reduction, which is discussed in more detail in Section 2.2. Despite the abundance of covalent protein complexes in this venom, they have not been reported to date.

### 2.2. Venom Proteome of N. nivea

An in-gel digestion approach was utilised to increase the overall coverage of protein identification given the additional separation afforded with SDS-PAGE. Gel bands from the reduced gels (Figure 2B) were pooled based on their position on the gel (see Supplementary Materials, Figure S1) and then analysed with tandem mass spectrometry. This analysis identified 240 proteins or proteoforms. Of these, nine could not be assigned to known proteins using all Serpent entries in the UniProt database (Table 1). These proteins were categorised by protein family and relative abundance is given. Relative abundance was calculated using normalized spectral abundance factors, as outlined by Deng et al. [36].

**Figure 3:**
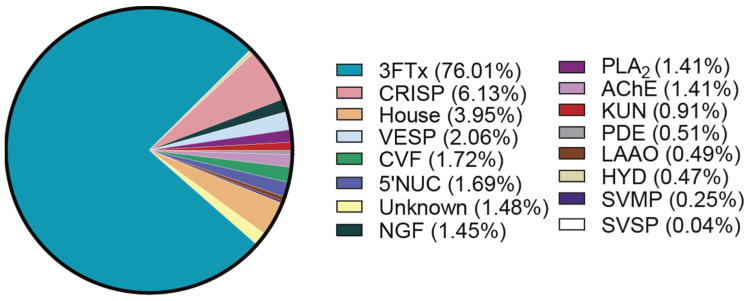
Global percentage of each protein family identified in *N. nivea* venom. Housekeeping proteins abbreviated to ‘House’.

By considering the relative abundance of each protein family, a global image of the *N. nivea* venom proteome can be constructed (Figure 3). As expected, based on its clinical presentation, previous reports, and SEC elution profile, *N. nivea* venom largely consists of low-MW proteins, specifically 76.01% 3FTx. A small proportion of PLA_2_ (1.41%) was identified, which marks the first report of PLA_2_ in *N. nivea* venom. While it is unusual for cobra venom to contain such a small PLA_2_ component, it is a phenomenon that has been reported in the venom of other non-spitting African cobras [37,38,39]. The second most prominent family of toxins was the CRISP (6.13%), which has previously been reported in much lower abundance [14]. The third most abundant proteins are housekeeping proteins (3.95%), which are often excluded from reports on venom proteomes but have been included here to give an accurate picture of toxin abundance in the crude venom mixture.

From the first proteomics analysis of *N. nivea* venom, Tan et al. provided an initial comparison to other snake species, particularly those in the same genus [14]. Interestingly, when compared with the proteome published by Tan et al., we see a much lower proportion of SVMP with these proteins, comprising only 0.25% compared with 6.79%, as previously reported [14]. This highlights the importance of conducting multiple proteomic studies on the same snake venom to gain a broader insight into any variations in the venom.

**Figure 4:**
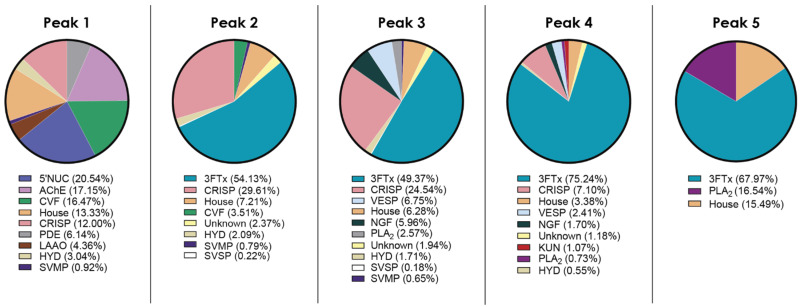
Percentage of each protein family identified in *N. nivea* venom grouped by SEC peak. Housekeeping proteins abbreviated to ‘House’. Each pie chart corresponds to labelled fractions from the SEC elution profile in Figure 1.

To gain insights into the composition of protein complexes in *N. nivea* venom, the proteomics results are grouped by SEC fraction (Figure 4). As expected, SEC peak 1 is made up of higher MW proteins, and the differences between the non-reduced and reduced gels for this peak can be largely attributed to the dissociation of the well-reported CVF protein complex [40]. Interestingly, when looking at SEC peak 2, we can see the presence of 3FTx, which has an average MW of 6 kDa. Based on its MW, this toxin should not be present in such an early eluting SEC fraction. However, as noted above, there is an unexpectedly low MW band present in both the non-reduced and reduced gels, which likely corresponds to these 3FTx. This needs to be investigated in the future to assess if this provides evidence of non-covalent complexes containing 3FTx or if it is an artifact of the SEC purification.

Peaks 3 and 4 contain proteins of the expected MW for this elution volume. Notably, the large increase in low-MW band intensity seen under reducing SDS-PAGE conditions for peak 4 (Figure 2) corresponds to the formation of a 3FTx or KUN monomer. This suggests that these small toxins form covalent protein complexes with other proteins in these fractions. Finally, looking at SEC peak 5, we see that the main toxin family present is 3FTx with some PLA_2_ toxins seen as well, which is unsurprising based on the low-MW species seen in the electrophoretic profiles. Here, we again see a difference in electrophoretic profile between the non-reduced and reduced gels (Figure 2), suggesting that there are covalent 3FTx or PLA_2_ complexes in *N. nivea* venom.

**Figure 5:**
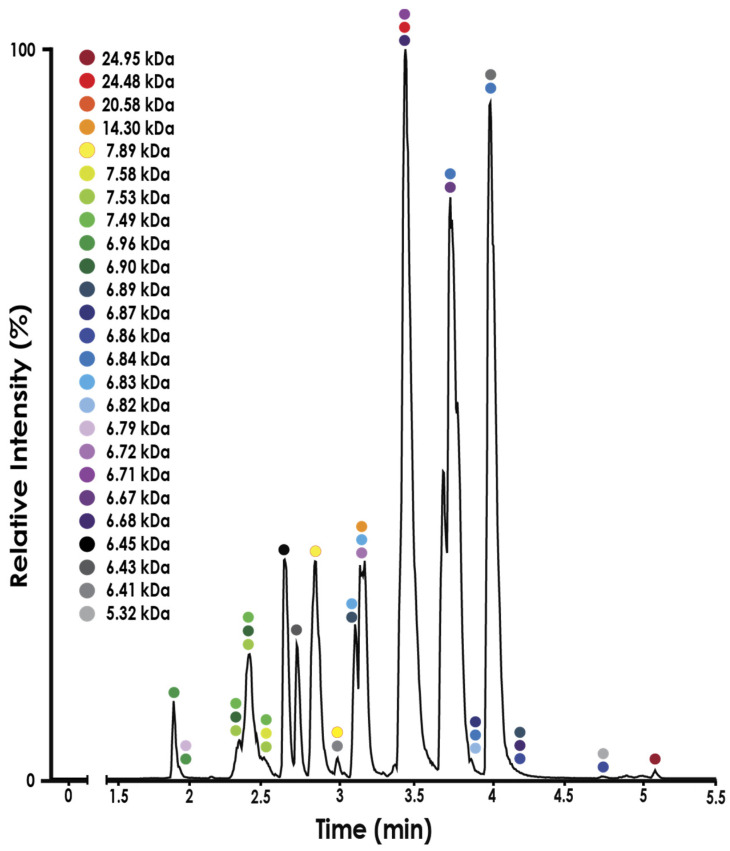
LC-MS chromatograph of a sample containing combined proteins from bands 19–22. Deconvoluted masses measured at each retention time are indicated by coloured markers.

To probe the possibility of covalent protein complexes within *N. nivea* venom, we conducted an LC-MS experiment to gain more detailed insights into the small-MW species in this venom. A pooled sample containing bands 19–22 was analysed using LC-MS under denaturing conditions to further separate these components and gain accurate masses. As expected, a large number of proteins or proteoforms were found (Figure 5). Of most interest was the species identified with a MW of 14.30 kDa (Figure S2). When looking at the average masses of all proteins found in bands 19–22 (Table S1), it is clear that no 14.30 kDa protein, or similar, was identified. In addition to this 14.30 KDa species, proteins with masses of 6.82 kDa and 7.49 kDa were also found in these fractions. Given this, we propose that this 14.30 kDa species is a covalent dimer of two different 3FTx. When compared to our proteomics data, it can be seen that these two masses fit well in the range of the average 3FTx masses identified (6.66 kDa–7.90 kDa). These data support our initial hypothesis that differences between non-reducing and reducing SDS PAGE gels highlight the presence of covalent protein complexes with *N. nivea* venom.

## 3. Conclusions and Future Directions

Here, we present a detailed view of the venom proteome of *N. nivea* using an in-gel bottom-up proteomics approach. It was found that the venom is dominated by 3FTx, making up 76.01% of the total venom proteome. Additionally, CRISPs, VESPs, CVFs, 5′NUCs, NGFs, PLA_2_s, AChEs, KUNs, PDEs, LAAOs, HYDs, SVMPs, and SVSP toxins were also identified in decreasing order of abundance. Contrary to previous reports of *N. nivea* venom, we did find PLA_2_ toxins, which highlights the importance of cataloguing the venom proteome of multiple snakes within a species to gain a full picture of intraspecies variation. We encourage further efforts to study the venom proteome of *N. nivea* to increase current understanding of this highly important and medically significant venom. Our current study marks the first report of covalent protein complexes in *N. nivea* venom. These complexes are of great interest due to increasing reports of synergic action increasing venom toxicity. Given the clinical manifestation of *N. nivea* venom, the large portion of 3FTx proteins, as well as the covalent 3FTx complex found, are of great interest as targets for alternative antivenoms. It is hence important to gain a greater understanding of these quaternary structures. Future efforts will be directed to the purification of these complexes for structure determination.

## 4. Materials and Methods

### 4.1. Materials

All reagents were purchased from Sigma Aldrich (St. Louis, MO, USA) unless otherwise specified.

Lyophilised pooled *N. nivea* venom was purchased from MToxins (Oshkosh, WI, USA) and was stored at −20 °C until required.

### 4.2. Chromatographic Separation

Venom proteins were fractioned with size exclusion chromatography (SEC). Lyophilized venom was reconstituted in 200 mM ammonium acetate (NH_4_OAc, pH 7.0) to a concentration of 25 mg/mL and loaded onto a Superdex200 10/300 size-exclusion column (GE Healthcare, Chicago, IL, USA) coupled to an ÄKTA Pure FPLC system (GE Healthcare). The column was equilibrated with 200 mM NH_4_OAc (400 μL) prior to sample loading (500 μL of 20 mg/mL stock). Fractions were collected (400 μL) at a flow rate of 0.4 mL/min with 200 mM NH_4_OAc as the eluant over a volume of 36 mL. Samples were stored at −20 °C until required.

### 4.3. One-Dimensional SDS-Polyacrylamide Gel Electrophoresis

Venom fractions were added in a 1:1 *v*/*v* ratio to 3 × reducing buffer (150 mM Tris-HCl, 300 mM dithiothreitol (DTT), 6% SDS, 30% glycerol, 0.3% bromophenol blue, pH 6.8) or 3 × non-reducing buffer (150 mM Tris-HCl, 6% SDS, 30% glycerol, 0.3% bromophenol blue, pH 6.8) and denatured at 95 °C for 15 min. Samples were then loaded onto a 4–15% Mini-Protean TGX Tris-HCl polyacrylamide gel (Bio-Rad, Hercules, CA, USA). Gel electrophoresis was performed with the following conditions: 140 V, 400 mA for 1 h in 1× SDS tris glycine running buffer. Precision Plus Protein Dual Colour standards (Bio-Rad, Hercules, CA, USA) were used as molecular weight markers. SDS-PAGE gels were visualised with Coomassie Brilliant Blue staining (Coomassie Brilliant Blue R250 dye, 10% (*v*/*v*) glacial acetic acid, 40% (*v*/*v*) methanol) prior to imaging using a ChemiDoc MP Imaging System (Bio-Rad, Hercules, CA, USA).

### 4.4. In-Gel Tryptic Digestion

Gel bands were exercised with a scalpel, cut into 1 mm^3^ pieces, and transferred to a microcentrifuge tube. Gel pieces were washed with 500 μL 20 mM ammonium bicarbonate (NH_4_HCO_3_) and incubated at 25 °C for 5 min before the NH_4_HCO_3_ was removed. Then, 400 μL of 50 mM NH_4_HCO_3_ 30% acetonitrile (can) was added and the mixture was sonicated for 15 min before pipetting off the solution. This step was repeated until the gel pieces were clear. The gel pieces were then incubated in 200 canACN at 25 °C for 15 min before being dried in a vacuum centrifuge. Then, 50 μL 10 mM DTT in 100 mM NH_4_HCO_3_ was added, and the gel pieces were incubated for 45 min at 56 °C. After incubation, 200 μL ACN was added, and the samples were incubated for a further 15 min before pipetting off the solution. Then, 50 μL of 55 mM iodoacetamide in 100 NH_4_HCO_3_ was added, and the samples were incubated for 30 min at 25 °C in darkness and the solution was removed via pipetting. A total of 100 μL of 5 mM NH_4_HCO_3_ was added, and the samples were incubated at 25 °C for 10 min before pipetting off the solution. Then, 200 μL ACN was added followed by a further 15 min incubation at 25 °C. The gel pieces were dried in a vacuum centrifuge and then suspended in 10 μL trypsin (10 ng/μL in 5 mM NH_4_HCO_3_). After 15 min at 25 °C, 10 μL of 5 mM NH_4_HCO_3_ in 20% ACN was added, and the samples were incubated at 37 °C for 18 hr. Then, 20 μL 1% formic acid was added, and the samples were sonicated for 15 min before transferring the liquid into the final microcentrifuge tube. The gel pieces were then further washed with 50 μL of 1% formic accanin ACN with sonicating for 15 min and the solution was transferred into the final sample tube. Finally, the gel pieces were washed with 100 can 100% ACN with sonicating, and that solution was added to the final sample tube. The combined solutions were concentrated to a volume of ~1 μL in a vacuum centrifuge and stored at −20 °C until future use.

### 4.5. LC-MS/MS Analysis

Peptides were separated using an UltiMate™3000 RSLC nano liquid chromatography system (ThermoFischer Scientific, Waltham, MA, USA) coupled online to a timsTOF Pro 2 mass spectrometer (Bruker Daltonics, Billerica, MA, USA). Peptide separation was performed using a 25 cm, 75 μm, 120 Å, ID Aurora C18 nano column with integrated emitter (Ion Opticks, Fitzroy, Australia) at a flow rate of 0.4 μL/min. The peptides (~200 ng) were eluted using a linear gradient of 2% to 25% Solvent B over 7.5 min, 25% to 37% Solvent B over 7.5 min, and 37% to 95% Solvent B over 3 min. This was followed by a 5 min wash with 95% Solvent B, and then a 5 min equilibration process with 5% Solvent B. (Solvent A: 0.1% (*v*/*v*) formic acid in water. Solvent B: 0.1% (*v*/*v*) formic acid in acetonitrile.) LC-MS/MS acquisition was performed using default parameters of the Data Independent Acquisition-Parallel Accumulation Serial Fragmentation (DIA-PASEF) mode. The conditions used were as follows: *m*/*z* range, 100–1700; polarity, positive; scan mode, DIA-PASEF; and TIMS ramp time, 100 ms using 100% duty cycle. Collision energy was increased linearly from 20 (0.6 V.s cm^2^) to 59 eV (1.6 V.s cm^2^).

### 4.6. MS/MS Data Analysis

MS data were analysed using PEAKS Studio 11 searching against all Serpent entries in the UniProt database (accession date: 7 May 2023). Parameters for the search were as follows: Tryptic peptides were set at a maximum of 3 missed cleavages and semi-specific digestion mode. A peptide mass tolerance of 10 ppm and fragment ion tolerance set at 0.02 Da. Maximum allowed variable PTMs per peptide was set at 3 using PEAKS PTM built-in modifications. The peptide length was set as 6–45 and report filtered by protein −10 logP ≥ 15 and unique peptides of ≥1. After database matching, proteins with non-toxic effects were denoted as ‘housekeeping’.

### 4.7. Estimation of Protein Relative Abundance

Relative abundance was estimated using normalised spectral abundance factors (NSAF) as outlined by Deng et al. [36]. In short, the spectral abundance (SAF) of each protein is calculated by dividing its spectral counts by its length. Each SAF is then divided by the sum of all SAFs to give the NSAF. This value was then multiplied by 100 to yield relative abundance as a percentage.

### 4.8. LC-MS Analysis of Venom Fractions

Venom samples were reconstituted in 5% acetonitrile (ACN) and 0.1% formic acid (FA) and then analysed using an I-Class Acquity UPLC system coupled to a Xevo G2-XS Q-ToF mass spectrometer (Waters Corporation, Milford, CT, USA). The sample (2 μg) was loaded onto an Acquity UPLC Protein BEH C4 (300 Å pore size, 1.7 μm particle size, 2.1 mm ID × 50 mm bed length) column (Waters Corporation, Milford, USA) at a flow rate of 0.3 mL/min. The gradient for Solvent B used was as follows: 5% to 15 % over 1 min, 15% to 55% over 8 min, 55% to 95% over 1 min, held at 95% over 1 min, 95% to 5% over 1 min, and held at 5% for 2 min (Solvent A: 99.9% MS-grade water/0.1% FA (*v*/*v*). Solvent B: 99.9% ACN/0.1% FA (*v*/*v*)). The column temperature was maintained at 80 °C. The mass spectrometer instrument conditions as follows: *m*/*z* range, 250–5000; polarity, positive; analyser mode, sensitivity; capillary voltage, 3.5 kV; sampling cone voltage, 80 V; source temperature, 120 °C; desolvation temperature, 300 °C; desolvation gas flow, 800 L/h; source offset, 90; and quadrupole profile, 500, 1000, and 1500. All masses were manually deconvoluted using ESIprot [41].

## Figures and Tables

**Table 1:** Protein families identified from *N. nivea* venom fractions using nanoLC-MS/MS. Relative abundance is calculated using the normalized spectral abundance factor. Results are grouped by gel band.

Protein Family	Number of Proteins	R.A (%)
Band 1		
PDE	3	0.41
Band 2		
AChE	5	0.26
PDE	2	0.08
Band 3		
CVF	7	0.22
5′NUC	3	0.66
LAAO	4	0.35
AChE	14	0.90
SVMP	2	0.07
Band 4		
CVF	7	0.49
5′NUC	3	1.02
LAAO	1	0.13
AChE	2	0.24
Band 5		
CVF	3	0.24
House	4	0.12
Band 6		
CVF	1	0.06
Band 7		
CVF	6	0.70
Band 8		
No hits > 1 unique pep	-	-
Band 9		
SVMP	1	0.12
Unknown	3	0.32
Band 10		
SVMP	2	0.03
3FTx	6	2.97
SVSP	2	0.04
Housekeeping	6	0.16
Unknown	4	0.14
Band 11		
CRISP	7	0.99
HYD	1	0.25
Housekeeping	9	0.97
Band 12		
CRISP	11	4.76
HYD	1	0.16
Housekeeping	1	0.14
Band 13		
CRISP	1	0.15
Band 14		
VESP	6	0.48
NGF	3	0.85
CRISP	1	0.08
Band 15		
VESP	2	1.16
NGF	2	0.60
PLA_2_	2	0.63
3FTx	2	1.24
Housekeeping	3	0.09
Band 16		
3FTx	5	7.82
Housekeeping	4	0.12
Band 17		
3FTx	13	31.15
KUN	1	0.39
Housekeeping	4	0.18
Band 18		
VESP	5	0.42
CRISP	2	0.15
HYD	1	0.6
Housekeeping	5	0.27
Band 19		
3FTx	13	29.60
KUN	1	0.52
Housekeeping	14	1.10
Unknown	2	1.01
Band 20		
No hits > 1 unique pep	-	-
Band 21		
PLA_2_	4	0.79
Housekeeping	13	0.55
Band 22		
3FTx	4	3.23
Housekeeping	6	0.19

## Data Availability

The data are contained within the manuscript.

## Supplementary Materials

The following supporting information can be downloaded at: https://www.mdpi.com/article/10.3390/toxins16020063/s1, Figure S1: Annotated gels showing bands that were combined for in-gel digestion.; Figure S2: Mass spectrum extracted from the LC-MS chromatogram at 3.24 min; Table S1: Proteins identified in *N. nivea* venom fractions using LC-MS/MS. Relative abundance is calculated using normalized spectral abundance factors, as outlined in the Methods.
