# Supplementary material for: Proteomic Investigation of Cape Cobra (Naja nivea) Venom Reveals First Evidence of Quaternary Protein Structures
Source: Toxins (Basel). 2024 Jan 23;16(2):63. doi: 10.3390/toxins16020063 (PMC10892407; doi:10.3390/toxins16020063)
Supplement: Supplementary file 1 [file toxins-16-00063-s001.zip › toxins-2771610-supplementary.pdf]

Supplementary material Proteomic Investigation of Cape Cobra (*Naja nivea*) Venom Reveals First Evidence of Quaternary Protein Structures

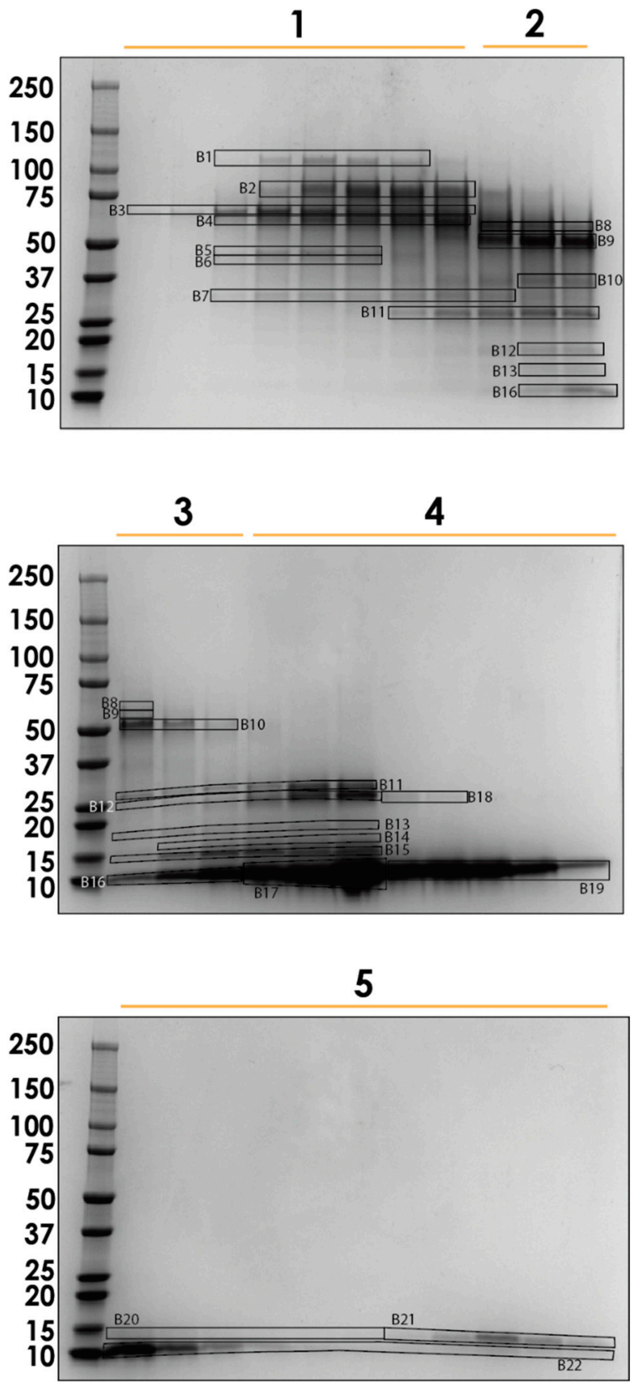

Figure S1: Annotated gels showing bands that were combined for in-gel digestion.

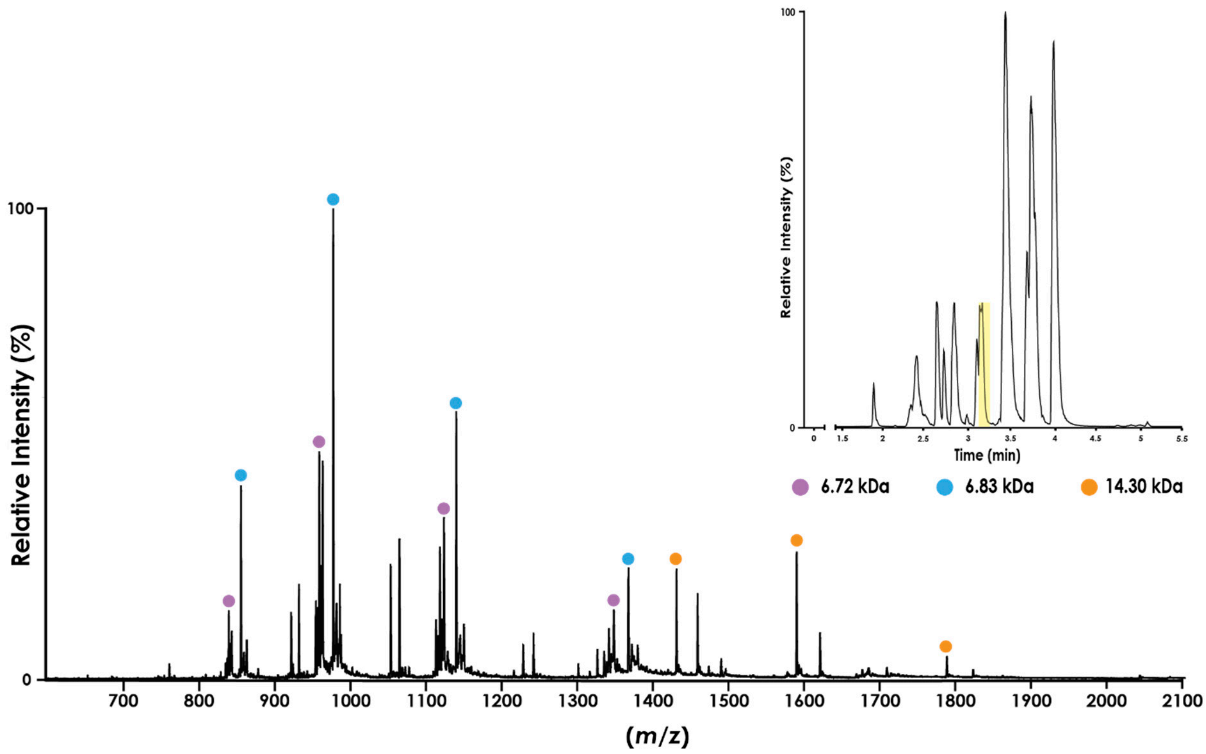

Figure S2: Mass spectrum extracted from the chromatogram at 3.24 min. Charge states for each species of interest are denoted by coloured circles, and their corresponding deconvoluted mass is given.

Table S1: Proteins identified from *N. nivea* venom fractions using LC-MS/MS. Relative abundance is calculated using normalised spectral abundance factors as outlined in the methods.

Band 1							
Accession	Unique peptides	Spectral count	length	SAF	Relavtive abundance (%)	protein family	Avg. Mass
A0A2D0TC04	3	16	830	0.019277108	0.14	PDE	94616
A0A8C6X0L1	3	16	853	0.018757327	0.14	PDE	96989
A0A8C6V7L7	3	16	889	0.01799775	0.13	PDE	101012
Band 2							
Accession	Unique peptides	Spectral count					Avg. Mass
R4FKE6	5	4	566	0.007067138	0.05	AChE	63242
A0A670ZA06	5	4	588	0.006802721	0.05	AChE	65198
A0A8C6Y6V0	5	5	547	0.009140768	0.07	AChE	60323
A0A8C7E5F6	3	5	581	0.008605852	0.06	AChE	65240
A0A8C5S3L4	3	3	655	0.004580153	0.03	AChE	73567
A0A2D0TC04	2	5	830	0.006024096	0.04	PDE	94616
A0A8C6X0L1	2	5	853	0.005861665	0.04	PDE	96989
Band 3							
Accession	Unique peptides	Spectral count					Avg. Mass
Q91132	10	18	1642	0.010962241	0.08	CVF	184517
A0A2I4HXH5	9	16	529	0.030245747	0.23	5'NUC	58198
A0A8C6XAL5	3	16	568	0.028169014	0.21	5'NUC	62399
A0A8C7DVS3	3	16	517	0.030947776	0.23	5'NUC	56646
A0A8C6V9N4	3	12	514	0.023346304	0.17	LAAO	58792
A0A670ZA06	3	9	588	0.015306122	0.11	AChE	65198
A0A8C6Y6V0	3	9	547	0.016453382	0.12	AChE	60323
A0A8C7E5F6	3	9	581	0.015490534	0.12	AChE	65240
Q92035	3	6	606	0.00990099	0.07	AChE	68074
A0A8C5S3L4	2	6	655	0.009160305	0.07	AChE	73567
R4FJM1	2	6	545	0.011009174	0.08	AChE	60425
D3TTC1	2	3	593	0.005059022	0.04	SVMP	66292
D6PXE8	2	3	593	0.005059022	0.04	SVMP	66246
A0A0A1WCE4	2	3	513	0.005847953	0.04	AChE	57649
A0A023VVX3	2	3	513	0.005847953	0.04	AChE	57630
A0A098LYB5	2	3	576	0.005208333	0.04	AChE	64762
A0A346CLZ4	2	3	605	0.004958678	0.04	AChE	68520
A0A098LYD4	2	3	605	0.004958678	0.04	AChE	68695
A0A023VWS2	2	3	608	0.004934211	0.04	AChE	68986
A0A0A1WDT1	2	3	608	0.004934211	0.04	AChE	69005
R4FID0	2	3	542	0.005535055	0.04	LAAO	61568
A0A8C7E3Y2	2	4	1179	0.003392706	0.03	CVF	132933
A0A8C6Y1E6	2	4	1203	0.003325021	0.02	CVF	135417
A0A8C6Y1Y9	2	4	1215	0.003292181	0.02	CVF	136664

A0A8C6Y3T6	2	4	1221	0.003276003	0.02	CVF	137551
A0A8C6Y2G7	2	4	1240	0.003225806	0.02	CVF	139848
A0A8C7E4H5	2	4	1254	0.003189793	0.02	CVF	141014
A8QL52	2	5	517	0.00967118	0.07	LAAO	58764
A8QL51	2	5	517	0.00967118	0.07	LAAO	58811
A0A098LY86	2	3	401	0.007481297	0.06	AChE	45999
Band 4							
Accession	Unique peptides	Spectral count					Avg. Mass
Q91132	13	28	1642	0.017052375	0.13	CVF	184517
A0A2I4HXH5	6	24	529	0.04536862	0.34	5'NUC	58198
A0A8C6XAL5	4	25	568	0.044014085	0.33	5'NUC	62399
A0A8C7DVS3	4	25	517	0.048355899	0.36	5'NUC	56646
A0A8C6V9N4	2	9	514	0.017509728	0.13	LAAO	58792
A0A8C6Y6V0	2	9	547	0.016453382	0.12	AChE	60323
A0A8C7E5F6	2	9	581	0.015490534	0.12	AChE	65240
A0A8C7E3Y2	2	10	1179	0.008481764	0.06	CVF	132933
A0A8C6Y1E6	2	10	1203	0.008312552	0.06	CVF	135417
A0A8C6Y1Y9	2	10	1215	0.008230453	0.06	CVF	136664
A0A8C6Y3T6	2	10	1221	0.008190008	0.06	CVF	137551
A0A8C6Y2G7	2	10	1240	0.008064516	0.06	CVF	139848
A0A8C7E4H5	2	10	1254	0.007974482	0.06	CVF	141014
Band 5							
Accession	Unique peptides	Spectral count					Avg. Mass
Q91132	2	15	1642	0.009135201	0.07	CVF	184517
A0A8C6Y1Y9	2	14	1215	0.011522634	0.09	CVF	136664
A0A8C7E4H5	2	14	1254	0.011164274	0.08	CVF	141014
V8NRX1	2	2	462	0.004329004	0.03	house	51876
A0A8C6X610	2	2	483	0.004140787	0.03	house	53183
A0A6B2F5Z9	2	2	486	0.004115226	0.03	house	53578
A0A8C5S523	2	2	460	0.004347826	0.03	house	50716
Band 6							
Accession	Unique peptides	Spectral count					Avg. Mass
Q91132	2	14	1642	0.008526188	0.06	CVF	184517
Band 7							
Accession	Unique peptides	Spectral count					Avg. Mass
Q91132	3	21	1642	0.012789281	0.10	CVF	184517
A0A8C6Y1E6	3	20	1203	0.016625104	0.12	CVF	135417
A0A8C6Y1Y9	3	20	1215	0.016460905	0.12	CVF	136664
A0A8C6Y2G7	3	20	1240	0.016129032	0.12	CVF	139848
A0A8C7E4H5	3	20	1254	0.015948963	0.12	CVF	141014
A0A8C7E3Y2	3	19	1179	0.016115352	0.12	CVF	132933

Band 8								
no hits > 1 unique pep								
Band 9								
Accession	Unique peptides	Spectral count						Avg. Mass
D3TTC2	2	10	613	0.016313214	0.12	SVMP		69181
A0A8C6XIQ9	2	9	614	0.01465798	0.11	unknown		68844
A0A8C6XLA9	2	9	614	0.01465798	0.11	unknown		69048
A0A8C6XJV4	2	9	614	0.01465798	0.11	unknown		68942
Band 10								
Accession	Unique peptides	Spectral count						Avg. Mass
D3TTC2	2	3	613	0.004893964	0.04	SVMP		69181
A0A8C6XIT1	2	3	614	0.004885993	0.04	unknown		68936
A0A8C6XIQ9	2	3	614	0.004885993	0.04	unknown		68844
A0A8C6XLA9	2	3	614	0.004885993	0.04	unknown		69048
A0A8C6XJV4	2	3	614	0.004885993	0.04	unknown		68942
A0A6B2F5Z9	2	2	486	0.004115226	0.03	house		53578
V8NRX1	2	2	462	0.004329004	0.03	house		51876
A0A8C6X610	2	2	483	0.004140787	0.03	house		53183
A0A670Z4J3	2	3	508	0.005905512	0.04	house		55607
P01456	2	4	60	0.066666667	0.50	3FTx		6697
P01455	2	4	60	0.066666667	0.50	3FTx		6696
A0A8C6Y570	2	1	317	0.003154574	0.02	SVSP		34594
A0A670YN01	2	1	356	0.002808989	0.02	SVSP		38378
A0A8C6XBU8	2	1	454	0.002202643	0.02	house		50316
P01462	2	4	60	0.066666667	0.50	3FTx		6858
P01464	2	4	60	0.066666667	0.50	3FTx		6856
P01465	2	4	60	0.066666667	0.50	3FTx		6857
P01466	2	4	60	0.066666667	0.50	3FTx		6856
A0A8C6X0T5	2	1	149	0.006711409	0.05	house		15926
D5LMJ3	2	1	607	0.001647446	0.01	SVMP		68254
Band 11								
Accession	Unique peptides	Spectral count						Avg. Mass
A0A8C5RUN6	5	9	268	0.03358209	0.25	HYD		30480
A0A8C6X9A7	5	9	266	0.033834586	0.25	house		30534
A0A670Z4J3	4	7	508	0.013779528	0.10	house		55607
A0A8C6XYH4	2	5	193	0.025906736	0.19	house		22033
A0A8C6XXU9	2	5	239	0.020920502	0.16	CRISP		26755
A0A8C6XZL9	2	5	239	0.020920502	0.16	CRISP		26822
A0A8C6XXV5	2	5	239	0.020920502	0.16	CRISP		26854
A0A6B2F5Z9	2	6	486	0.012345679	0.09	house		53578

V8NCE3	2	5	450	0.011111111	0.08	house	51067
A0A8C6XBU8	2	3	454	0.00660793	0.05	house	50316
Q7T1K6	2	5	239	0.020920502	0.16	CRISP	26882
F2Q6F2	2	4	239	0.016736402	0.12	CRISP	26688
A0A223PK48	2	4	239	0.016736402	0.12	CRISP	27351
A0A223PK22	2	4	257	0.015564202	0.12	CRISP	29388
J9ZZN7	2	3	247	0.012145749	0.09	house	28165
A0A6I9XF37	2	3	251	0.011952191	0.09	house	28634
V8N8G6	2	1	360	0.002777778	0.02	house	40263
Band 12							
Accession	Unique peptides	Spectral count					Avg. Mass
A0A8C6Y1Y2	4	26	239	0.108786611	0.81	CRISP	26985
Q7T1K6	4	24	239	0.10041841	0.75	CRISP	26882
Q7ZT98	4	22	239	0.092050209	0.69	CRISP	26869
A0A8C6XYH4	4	8	193	0.041450777	0.31	CRISP	22033
A0A8C6XXU9	3	17	239	0.071129707	0.53	CRISP	26755
A0A8C6XZL9	3	17	239	0.071129707	0.53	CRISP	26822
A0A8C6XXV5	3	17	239	0.071129707	0.53	CRISP	26854
A0A8C5RUN6	3	6	268	0.02238806	0.17	HYD	30480
A0A8C6X9A7	3	5	266	0.018796992	0.14	house	30534
C1JZW4	2	5	237	0.021097046	0.16	CRISP	26305
F2Q6G2	2	5	238	0.021008403	0.16	CRISP	26458
F2Q6G3	2	5	238	0.021008403	0.16	CRISP	26443
V8NKT2	2	5	244	0.020491803	0.15	CRISP	27268
Band 13							
Accession	Unique peptides	Spectral count					Avg. Mass
A0A8C6XYH4	4	4	193	0.020725389	0.15	CRISP	22033
Band 14							
Accession	Unique peptides	Spectral count					Avg. Mass
P61899	2	8	116	0.068965517	0.51	NGF	13064
Q5YF89	2	8	241	0.033195021	0.25	NGF	27030
P82885	2	2	108	0.018518519	0.14	VESP	12038
P83234	2	2	190	0.010526316	0.08	CRISP	21174
A0A8C6VC33	2	2	204	0.009803922	0.07	VESP	22638
A0A182C6D0	2	2	213	0.009389671	0.07	VESP	23498
A0A098LWX5	2	2	216	0.009259259	0.07	VESP	24116
A0A8C5RJP3	2	2	218	0.009174312	0.07	VESP	24380
V8NEU2	2	2	220	0.009090909	0.07	VESP	24506
V9I168	2	3	243	0.012345679	0.09	NGF	27284
Band 15							
Accession	Unique peptides	Spectral count					Avg. Mass

P82885	9	11	108	0.101851852	0.76	VESP	12038
A0A8C6VC33	9	11	204	0.053921569	0.40	VESP	22638
P61899	3	6	116	0.051724138	0.39	NGF	13064
Q5YF89	3	7	241	0.029045643	0.22	NGF	27030
P00601	3	5	119	0.042016807	0.31	PLA2	13360
P00600	2	5	119	0.042016807	0.31	PLA2	13427
V8NRX1	2	2	462	0.004329004	0.03	house	51876
A0A8C6X610	2	2	483	0.004140787	0.03	house	53183
A0A6B2F5Z9	2	2	486	0.004115226	0.03	house	53578
P01456	2	5	60	0.083333333	0.62	3FTx	6697
P01455	2	5	60	0.083333333	0.62	3FTx	6696
Band 16							
Accession	Unique peptides	Spectral count					Avg. Mass
P01456	3	9	60	0.15	1.12	3FTx	6697
P01455	3	9	60	0.15	1.12	3FTx	6696
V8NRX1	3	2	462	0.004329004	0.03	house	51876
A0A8C6X610	3	2	483	0.004140787	0.03	house	53183
A0A6B2F5Z9	3	2	486	0.004115226	0.03	house	53578
A0A670Z4J3	2	2	508	0.003937008	0.03	house	55607
P01462	2	15	60	0.25	1.86	3FTx	6858
P01464	2	15	60	0.25	1.86	3FTx	6856
P01465	2	15	60	0.25	1.86	3FTx	6857
Band 17							
Accession	Unique peptides	Spectral count					Avg. Mass
P01453	4	37	60	0.616666667	4.59	3FTx	6682
P01456	3	39	60	0.65	4.84	3FTx	6697
P01390	3	7	71	0.098591549	0.73	3FTx	7902
P01462	2	40	60	0.666666667	4.96	3FTx	6858
P01465	2	40	60	0.666666667	4.96	3FTx	6857
P01463	2	38	60	0.633333333	4.72	3FTx	6871
P25674	2	7	71	0.098591549	0.73	3FTx	7821
P01458	2	11	60	0.183333333	1.36	3FTx	6791
P01400	2	6	65	0.092307692	0.69	3FTx	7430
P00986	2	3	57	0.052631579	0.39	KUN	6466
A0A6B2F5Z9	2	3	486	0.00617284	0.05	house	53578
V8NRX1	2	3	462	0.006493506	0.05	house	51876
A0A8C6X610	2	3	483	0.00621118	0.05	house	53183
P01460	2	11	60	0.183333333	1.36	3FTx	6812
P01459	2	11	60	0.183333333	1.36	3FTx	6839
A0A670Z4J3	2	3	508	0.005905512	0.04	house	55607
P01421	2	1	61	0.016393443	0.12	3FTx	6913
P0C547	2	2	21	0.095238095	0.71	3FTx	2400

Band 18							
Accession	Unique peptides	Spectral count					Avg. Mass
P82885	2	2	108	0.018518519	0.14	VESP	12038
P83234	2	2	190	0.010526316	0.08	CRISP	21174
A0A8C6VC33	2	2	204	0.009803922	0.07	VESP	22638
A0A182C6D0	2	2	213	0.009389671	0.07	CRISP	23498
A0A098LWX5	2	2	216	0.009259259	0.07	VESP	24116
A0A8C5RJP3	2	2	218	0.009174312	0.07	VESP	24380
V8NEU2	2	2	220	0.009090909	0.07	VESP	24506
A0A8C6X9A7	2	2	266	0.007518797	0.06	house	30534
A0A8C5RUN6	2	2	268	0.007462687	0.06	HYD	30480
A0A6J1W1H3	2	2	274	0.00729927	0.05	house	31377
U3FCT9	2	2	274	0.00729927	0.05	house	31577
A0A670ZLF7	2	2	274	0.00729927	0.05	house	31175
A0A6J1VT72	2	2	288	0.006944444	0.05	house	33047
Band 19							
Accession	Unique peptides	Spectral count					Avg. Mass
P01453	4	31	60	0.516666667	3.85	3FTx	6682
P01454	3	29	60	0.483333333	3.60	3FTx	6669
P01456	3	34	60	0.566666667	4.22	3FTx	6697
P01390	2	7	71	0.098591549	0.73	3FTx	7902
P01462	2	32	60	0.533333333	3.97	3FTx	6858
P01465	2	32	60	0.533333333	3.97	3FTx	6857
P01463	2	29	60	0.483333333	3.60	3FTx	6871
P01458	2	12	60	0.2	1.49	3FTx	6791
P25674	2	7	71	0.098591549	0.73	3FTx	7821
V8NTL3	2	4	126	0.031746032	0.24	house	13507
V8NSM1	2	4	129	0.031007752	0.23	house	13855
A0A8C6X0T5	2	4	149	0.026845638	0.20	house	15926
P00986	2	4	57	0.070175439	0.52	KUN	6466
P01400	2	6	65	0.092307692	0.69	3FTx	7430
A0A6B2F5Z9	2	2	486	0.004115226	0.03	house	53578
V8NRX1	2	2	462	0.004329004	0.03	house	51876
A0A8C6X610	2	2	483	0.004140787	0.03	house	53183
A0A8C6XFH6	2	6	81	0.074074074	0.55	unknown	9071
A0A0U5ARS4	2	5	72	0.069444444	0.52	3FTx	7959
A0A8C6XFM5	2	5	81	0.061728395	0.46	unknown	8945
P01460	2	9	60	0.15	1.12	3FTx	6812
P01459	2	9	60	0.15	1.12	3FTx	6839
D6RUZ0	2	2	233	0.008583691	0.06	house	25972
A0A8C5S8V5	2	2	332	0.006024096	0.04	house	37341
V8N8G6	2	2	360	0.005555556	0.04	house	40263

A0A8C6VAB1	2	2	375	0.005333333	0.04	house	41793
A0A670YBF6	2	2	375	0.005333333	0.04	house	41737
A0A8C5SDB1	2	2	378	0.005291005	0.04	house	42008
A0A6J1VWC1	2	2	389	0.005141388	0.04	house	43133
A0A8C6XB43	2	2	450	0.004444444	0.03	house	49843
Band 20							
no hits > 1 unique pep							
Band 21							
Accession	Unique peptides	Spectral count					Avg. Mass
Q5DK65	2	2	220	0.009090909	0.07	house	24743
Q5DK66	2	2	224	0.008928571	0.07	house	25203
A0A8C5S8V5	2	2	332	0.006024096	0.04	house	37341
V8N8G6	2	2	360	0.005555556	0.04	house	40263
U3FXK3	2	2	375	0.005333333	0.04	house	41737
A0A8C6VAB1	2	2	375	0.005333333	0.04	house	41793
A0A8C5SDB1	2	2	378	0.005291005	0.04	house	42008
A0A8C5SAH1	2	2	396	0.005050505	0.04	house	43793
A0A8C6XB43	2	2	450	0.004444444	0.03	house	49843
A0A6B2F5Z9	2	2	486	0.004115226	0.03	house	53578
V8NRX1	2	2	462	0.004329004	0.03	house	51876
A0A8C6X610	2	2	483	0.004140787	0.03	house	53183
A0A670Z4J3	2	3	508	0.005905512	0.04	house	55607
A0A8C6XKE6	2	3	109	0.027522936	0.20	PLA2	11921
A0A8C6XK26	2	3	109	0.027522936	0.20	PLA2	11992
A4FS04	2	3	119	0.025210084	0.19	PLA2	13188
P25498	2	3	119	0.025210084	0.19	PLA2	13229
Band 22							
Accession	Unique peptides	Spectral count					Avg. Mass
P01456	3	8	60	0.133333333	0.99	3FTx	6697
P01455	3	8	60	0.133333333	0.99	3FTx	6696
P01454	3	5	60	0.083333333	0.62	3FTx	6669
P01453	3	5	60	0.083333333	0.62	3FTx	6682
V8NRX1	3	2	462	0.004329004	0.03	house	51876
A0A8C6X610	2	2	483	0.004140787	0.03	house	53183
A0A6B2F5Z9	2	2	486	0.004115226	0.03	house	53578
A0A670Z4J3	2	2	508	0.003937008	0.03	house	55607
A0A8C5S523	2	2	460	0.004347826	0.03	house	50716
A0A8C5WTY5	2	2	463	0.004319654	0.03	house	51349
